# 2-(1,3-Dithian-2-yl)-1,3-dithiane-2-carbaldehyde

**DOI:** 10.1107/S1600536808042864

**Published:** 2008-12-20

**Authors:** Hoong-Kun Fun, Reza Kia, Annada C. Maity, Shyamaprosad Goswami

**Affiliations:** a X-ray Crystallography Unit, School of Physics, Universiti Sains Malaysia, 11800 USM, Penang, Malaysia; b Department of Chemistry, Bengal Engineering and Science University, Shibpur, Howrah 711 103, India

## Abstract

The asymmetric unit of the title compound, C_9_H_14_OS_4_, comprises two crystallographically independent mol­ecules with similar conformations. In each mol­ecule, an intra­molecular C—H⋯O hydrogen bond generates a six-membered ring, producing an *S*(6) ring motif. All of the six-membered dithia­cyclo­hexane rings adopt chair conformations. The crystal structure is stabilized by four inter­molecular C—H⋯O and one C—H⋯S inter­action.

## Related literature

For details of hydrogen-bond motifs, see: Bernstein *et al.* (1995 ▶). For ring puckering analysis, see: Cremer & Pople (1975 ▶). For related literature, see: Goswami & Maity (2008 ▶); Rubin & Gleiter (2000 ▶); Wasserman & Parr (2004 ▶).
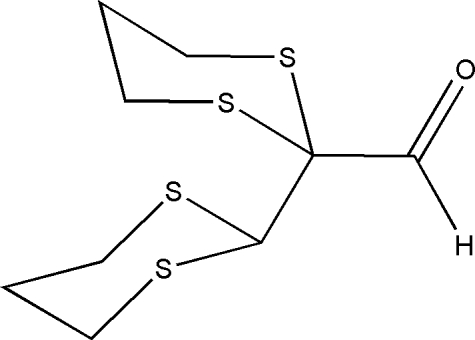


## Experimental

### 

#### Crystal data

C_9_H_14_OS_4_
*M* *_r_* = 266.44
Monoclinic, 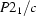

*a* = 13.0028 (2) Å
*b* = 13.6790 (2) Å
*c* = 13.4244 (2) Å
β = 91.873 (1)°
*V* = 2386.46 (6) Å^3^
*Z* = 8
Mo *K*α radiation
μ = 0.76 mm^−1^
*T* = 100.0 (1) K
0.39 × 0.28 × 0.19 mm

#### Data collection

Bruker SMART APEXII CCD area-detector diffractometer
Absorption correction: multi-scan (**SADABS**; Bruker, 2005 ▶) *T*_min_ = 0.754, *T*_max_ = 0.871
68856 measured reflections
12473 independent reflections
9371 reflections with *I* > 2σ(*I*)
*R*_int_ = 0.055

#### Refinement

*R*[*F* ^2^ > 2σ(*F* ^2^)] = 0.037
*wR*(*F* ^2^) = 0.087
*S* = 1.07
12473 reflections
253 parameters
H-atom parameters constrained
Δρ_max_ = 0.46 e Å^−3^
Δρ_min_ = −0.38 e Å^−3^

### 

Data collection: *APEX2* (Bruker, 2005 ▶); cell refinement: *SAINT* (Bruker, 2005 ▶); data reduction: *SAINT*; program(s) used to solve structure: *SHELXTL* (Sheldrick, 2008 ▶); program(s) used to refine structure: *SHELXTL*; molecular graphics: *SHELXTL*; software used to prepare material for publication: *SHELXTL* and *PLATON* (Spek, 2003 ▶).

## Supplementary Material

Crystal structure: contains datablocks global, I. DOI: 10.1107/S1600536808042864/is2376sup1.cif

Structure factors: contains datablocks I. DOI: 10.1107/S1600536808042864/is2376Isup2.hkl

Additional supplementary materials:  crystallographic information; 3D view; checkCIF report

## Figures and Tables

**Table 1:** Hydrogen-bond geometry (Å, °)

*D*—H⋯*A*	*D*—H	H⋯*A*	*D*⋯*A*	*D*—H⋯*A*
C2*A*—H2*AB*⋯O1*A*^i^	0.97	2.51	3.3530 (15)	146
C3*A*—H3*AB*⋯O1*A*	0.97	2.48	3.1024 (16)	122
C6*A*—H6*AB*⋯O1*B*^ii^	0.97	2.51	3.4292 (15)	159
C1*B*—H1*BA*⋯O1*B*	0.97	2.44	3.0508 (16)	121
C2*B*—H2*BA*⋯O1*B*^iii^	0.97	2.54	3.1913 (16)	124
C3*B*—H3*BA*⋯S2*A*^iv^	0.97	2.81	3.5932 (12)	138
C7*B*—H7*BA*⋯O1*A*^v^	0.97	2.54	3.3436 (17)	140

Symmetry codes: (i) 
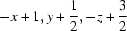
; (ii) 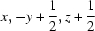; (iii) 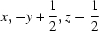; (iv) 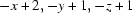; (v) 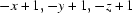.

**Fig. 1.**
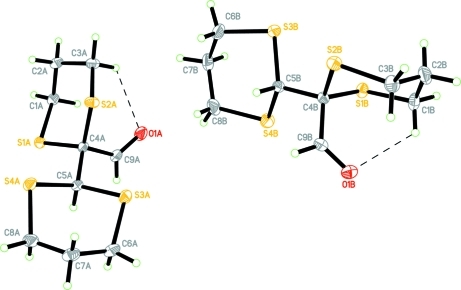
The molecular structure of (I), showing 50% probability displacement ellipsoids and the atomic numbering. Dashed lines show intramolecular hydrogen bonds.

**Fig. 2.**
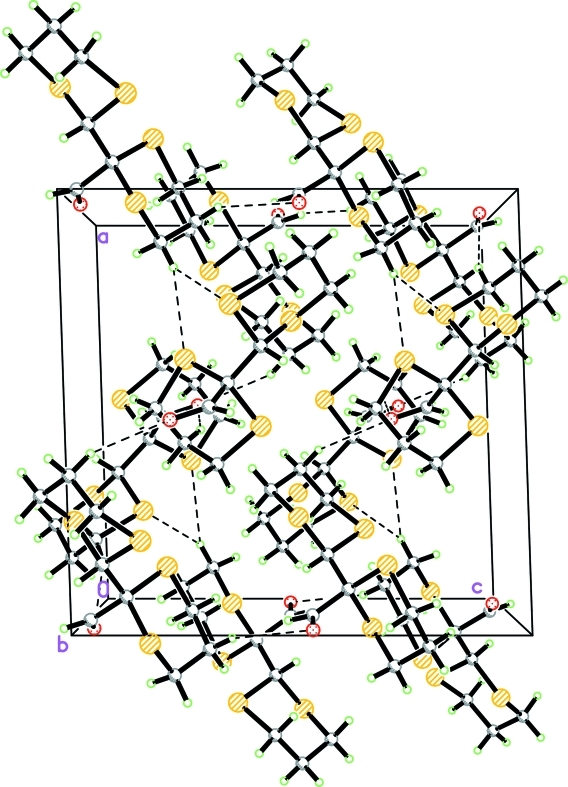
The crystal packing for (I), viewed down the b axis showing linking of molecules through C—H···O and C—H···S interactions. Intermolecular interactions are drawn as dashed lines.

## supplementary crystallographic information

### Comment

Vicinal tricarbonyl compounds are powerful electrophiles with widespread applications in organic synthesis (Rubin & Gleiter, 2000; Wasserman & Parr, 2004). They act as useful precursors to synthesis of elaborate heterocylic compounds and numerous novel biologically important substances such as FK-506, rapamycin and related immunosuppressants. They are also used to develop protease inhibitors derived from peptide carboxylic acids. Thioacetalization of carbonyl compounds (Goswami & Maity, 2008) plays an important role in organic synthesis. Dithioacetals have become widely used tools for C—C bond formation. Here we reported the first synthesis of 2,2'-bis(1,3-dithianyl)-2-carbaldehyde from the smallest vicinal tricarbonyl compound, 2-oxo-1,3-propandial.

In the title compound (I), Fig. 1, intramolecular C—H···O hydrogen bonds (Table 1) generate six-membered rings, producing *S*(6) ring motifs (Bernstein *et al.,* 1995). The S1A/C1A–C3A/S2A/C4A, S3A/C5A/S4A/C8A/C7A/C6A, S1B/C1B–C3B/S2B/C4B, and S3B/C5B/S4B/C8B/C7B/C6B rings adopt chair conformations with the ring puckering parameters (Cremer & Pople, 1975) of Q = 0.6979 (10) Å, Θ = 5.43 (8)°, Φ = 3.4 (9)°; Q = 0.7467 (10) Å, Θ = 171.28 (8)°, Φ = 246.6 (5)°; Q = 0.6967 (11) Å, Θ = 7.22 (9)°, Φ = 247.2 (7)°; Q = 0.7475 (11) Å, Θ = 170.82 (9)°, Φ = 248.2 (5)°, respectively. The crystal structure is stabilized by intermolecular C—H···O (× 4) and C—H···S interactions (Fig. 2).

### Experimental

To a stirred solution of 2-oxo-1,3-propandial (250 mg, 0.34 mmol) and boron trifluoride etherate (0.5 mL) in dichloromethane (50 mL) cooled at 0 °C is added propane dithiol (450 mg, 4.1 mmol) dropwise over 15 min with stirring. The mixture is stirred at room temperature for 3h. The progress of the reaction is monitored by TLC. After completion of the reaction, NaHCO_3_ solution is added slowly and carefully to neutralize the mixture at room temperature, which is then extracted with dichloromethane. The organic layer is dried (anhydrous Na_2_SO_4_) and then the solvent is removed under reduced pressure. The crude product was purified by column chromatography using silica gel with 20% ethyl acetate in petroleum ether as eluant to afford 2,2'-bis(1,3-dithianyl)-2-carbaldehyde (247 mg, 32%) as a colorless crystalline solid along with other thiane derivatives.

### Refinement

All of the hydrogen atoms were positioned geometrically with C—H = 0.93–0.98 Å and refined in the riding model approximation, with *U*_iso_(H) = 1.2*U*_eq_(C).

### Crystal data

**Table:**

C_9_H_14_OS_4_	*F*(000) = 1120
*M**_r_* = 266.44	*D*_x_ = 1.483 Mg m^−^^3^
Monoclinic, *P*2_1_/*c*	Mo *K*α radiation, λ = 0.71073 Å
Hall symbol: -P 2ybc	Cell parameters from 6037 reflections
*a* = 13.0028 (2) Å	θ = 2.6–35.5°
*b* = 13.6790 (2) Å	µ = 0.76 mm^−^^1^
*c* = 13.4244 (2) Å	*T* = 100 K
β = 91.873 (1)°	Block, colourless
*V* = 2386.46 (6) Å^3^	0.39 × 0.28 × 0.19 mm
*Z* = 8	

### Data collection

**Table:**

Bruker SMART APEXII CCD area-detector diffractometer	12473 independent reflections
Radiation source: fine-focus sealed tube	9371 reflections with *I* > 2σ(*I*)
graphite	*R*_int_ = 0.055
φ and ω scans	θ_max_ = 37.5°, θ_min_ = 1.6°
Absorption correction: multi-scan (*SADABS*; Bruker, 2005)	*h* = −22→20
*T*_min_ = 0.754, *T*_max_ = 0.871	*k* = −22→23
68856 measured reflections	*l* = −22→22

### Refinement

**Table:**

Refinement on *F*^2^	Primary atom site location: structure-invariant direct methods
Least-squares matrix: full	Secondary atom site location: difference Fourier map
*R*[*F*^2^ > 2σ(*F*^2^)] = 0.037	Hydrogen site location: inferred from neighbouring sites
*wR*(*F*^2^) = 0.087	H-atom parameters constrained
*S* = 1.07	*w* = 1/[σ^2^(*F*_o_^2^) + (0.0325*P*)^2^ + 0.5359*P*] where *P* = (*F*_o_^2^ + 2*F*_c_^2^)/3
12473 reflections	(Δ/σ)_max_ = 0.002
253 parameters	Δρ_max_ = 0.46 e Å^−^^3^
0 restraints	Δρ_min_ = −0.37 e Å^−^^3^

### Special details

**Table:**

Experimental. The low-temperature data was collected with the Oxford Cyrosystem Cobra low-temperature attachment.
Geometry. All esds (except the esd in the dihedral angle between two l.s. planes) are estimated using the full covariance matrix. The cell esds are taken into account individually in the estimation of esds in distances, angles and torsion angles; correlations between esds in cell parameters are only used when they are defined by crystal symmetry. An approximate (isotropic) treatment of cell esds is used for estimating esds involving l.s. planes.
Refinement. Refinement of F^2^ against ALL reflections. The weighted R-factor wR and goodness of fit S are based on F^2^, conventional R-factors R are based on F, with F set to zero for negative F^2^. The threshold expression of F^2^ > 2sigma(F^2^) is used only for calculating R-factors(gt) etc. and is not relevant to the choice of reflections for refinement. R-factors based on F^2^ are statistically about twice as large as those based on F, and R- factors based on ALL data will be even larger.

### Fractional atomic coordinates and isotropic or equivalent isotropic displacement parameters (Å^2^)

**Table:**

	*x*	*y*	*z*	*U*_iso_*/*U*_eq_	
S1A	0.46746 (2)	0.654917 (19)	0.92342 (2)	0.01556 (5)	
S2A	0.62228 (2)	0.66697 (2)	0.75806 (2)	0.01787 (5)	
S3A	0.74126 (2)	0.47975 (2)	0.86715 (2)	0.01958 (6)	
S4A	0.71041 (2)	0.65781 (2)	0.99797 (2)	0.02006 (6)	
O1A	0.48320 (8)	0.48599 (7)	0.72999 (8)	0.02660 (19)	
C1A	0.37902 (8)	0.68880 (8)	0.82162 (9)	0.01741 (19)	
H1AA	0.3555	0.6300	0.7875	0.021*	
H1AB	0.3194	0.7206	0.8487	0.021*	
C2A	0.42752 (9)	0.75689 (8)	0.74674 (9)	0.0190 (2)	
H2AA	0.3740	0.7823	0.7019	0.023*	
H2AB	0.4586	0.8118	0.7822	0.023*	
C3A	0.50888 (9)	0.70711 (9)	0.68583 (9)	0.0201 (2)	
H3AA	0.5305	0.7520	0.6347	0.024*	
H3AB	0.4781	0.6508	0.6526	0.024*	
C4A	0.56675 (8)	0.59204 (7)	0.85376 (8)	0.01396 (17)	
C5A	0.65016 (8)	0.55798 (7)	0.92955 (8)	0.01432 (17)	
H5AA	0.6159	0.5171	0.9784	0.017*	
C6A	0.80918 (9)	0.43427 (9)	0.97830 (10)	0.0229 (2)	
H6AA	0.7612	0.3980	1.0180	0.027*	
H6AB	0.8622	0.3891	0.9583	0.027*	
C7A	0.85868 (10)	0.51357 (9)	1.04276 (11)	0.0247 (2)	
H7AA	0.9051	0.5512	1.0027	0.030*	
H7AB	0.8993	0.4830	1.0961	0.030*	
C8A	0.78136 (10)	0.58263 (9)	1.08808 (10)	0.0238 (2)	
H8AA	0.8176	0.6252	1.1352	0.029*	
H8AB	0.7327	0.5443	1.1250	0.029*	
C9A	0.51184 (9)	0.50023 (8)	0.81473 (9)	0.0182 (2)	
H9AA	0.4995	0.4511	0.8607	0.022*	
S1B	0.86151 (2)	0.28160 (2)	0.29985 (2)	0.01813 (6)	
S2B	1.02461 (2)	0.43886 (2)	0.33386 (2)	0.01876 (6)	
S3B	0.78758 (2)	0.51420 (2)	0.34283 (2)	0.01985 (6)	
S4B	0.75193 (2)	0.35947 (2)	0.49927 (2)	0.02279 (6)	
O1B	1.00970 (7)	0.22837 (7)	0.47436 (7)	0.02534 (18)	
C1B	0.96908 (9)	0.21483 (9)	0.24967 (10)	0.0238 (2)	
H1BA	0.9986	0.1732	0.3017	0.029*	
H1BB	0.9432	0.1727	0.1964	0.029*	
C2B	1.05386 (10)	0.27878 (11)	0.20949 (10)	0.0276 (3)	
H2BA	1.0246	0.3201	0.1571	0.033*	
H2BB	1.1049	0.2371	0.1799	0.033*	
C3B	1.10724 (9)	0.34340 (10)	0.28785 (10)	0.0239 (2)	
H3BA	1.1672	0.3732	0.2593	0.029*	
H3BB	1.1308	0.3030	0.3434	0.029*	
C4B	0.92466 (8)	0.36320 (7)	0.38847 (8)	0.01385 (17)	
C5B	0.84575 (8)	0.43202 (8)	0.43385 (8)	0.01527 (18)	
H5BA	0.8826	0.4723	0.4837	0.018*	
C6B	0.71805 (10)	0.58963 (9)	0.42935 (10)	0.0251 (2)	
H6BA	0.7675	0.6215	0.4742	0.030*	
H6BB	0.6818	0.6403	0.3919	0.030*	
C7B	0.64127 (10)	0.53471 (10)	0.49091 (10)	0.0256 (2)	
H7BA	0.5930	0.5012	0.4462	0.031*	
H7BB	0.6026	0.5816	0.5290	0.031*	
C8B	0.68984 (11)	0.46041 (11)	0.56230 (10)	0.0275 (3)	
H8BA	0.6370	0.4344	0.6043	0.033*	
H8BB	0.7404	0.4932	0.6052	0.033*	
C9B	0.98274 (9)	0.31267 (8)	0.47398 (9)	0.01764 (19)	
H9BA	0.9988	0.3497	0.5305	0.021*	

### Atomic displacement parameters (Å^2^)

**Table:**

	*U*^11^	*U*^22^	*U*^33^	*U*^12^	*U*^13^	*U*^23^
S1A	0.01482 (11)	0.01624 (11)	0.01573 (12)	0.00201 (8)	0.00224 (9)	−0.00020 (9)
S2A	0.01485 (11)	0.02094 (12)	0.01797 (13)	0.00106 (9)	0.00283 (9)	0.00538 (9)
S3A	0.01766 (12)	0.01907 (12)	0.02204 (14)	0.00508 (9)	0.00125 (10)	−0.00104 (10)
S4A	0.02219 (13)	0.01382 (11)	0.02366 (14)	0.00037 (9)	−0.00701 (10)	−0.00098 (9)
O1A	0.0337 (5)	0.0184 (4)	0.0268 (5)	0.0044 (3)	−0.0117 (4)	−0.0054 (3)
C1A	0.0144 (4)	0.0167 (5)	0.0210 (5)	0.0019 (3)	−0.0002 (4)	0.0004 (4)
C2A	0.0185 (5)	0.0158 (5)	0.0226 (5)	0.0031 (4)	−0.0013 (4)	0.0032 (4)
C3A	0.0206 (5)	0.0216 (5)	0.0180 (5)	0.0030 (4)	0.0008 (4)	0.0063 (4)
C4A	0.0146 (4)	0.0122 (4)	0.0152 (5)	0.0007 (3)	0.0017 (3)	0.0002 (3)
C5A	0.0140 (4)	0.0120 (4)	0.0170 (5)	0.0001 (3)	0.0004 (3)	0.0008 (3)
C6A	0.0200 (5)	0.0180 (5)	0.0306 (6)	0.0042 (4)	−0.0018 (4)	0.0039 (4)
C7A	0.0199 (5)	0.0217 (5)	0.0320 (7)	0.0011 (4)	−0.0078 (5)	0.0051 (5)
C8A	0.0246 (5)	0.0226 (5)	0.0236 (6)	0.0005 (4)	−0.0089 (4)	0.0013 (4)
C9A	0.0180 (5)	0.0137 (4)	0.0229 (5)	0.0016 (3)	−0.0010 (4)	−0.0010 (4)
S1B	0.01571 (11)	0.01880 (12)	0.01966 (13)	0.00048 (9)	−0.00272 (9)	−0.00554 (9)
S2B	0.01721 (11)	0.01821 (12)	0.02107 (13)	−0.00308 (9)	0.00389 (10)	0.00171 (9)
S3B	0.02317 (13)	0.01821 (12)	0.01821 (13)	0.00516 (9)	0.00150 (10)	0.00249 (9)
S4B	0.02262 (13)	0.02165 (13)	0.02473 (15)	0.00012 (10)	0.01028 (11)	0.00316 (11)
O1B	0.0301 (5)	0.0229 (4)	0.0227 (4)	0.0064 (3)	−0.0031 (4)	0.0032 (3)
C1B	0.0233 (5)	0.0241 (6)	0.0239 (6)	0.0056 (4)	−0.0001 (4)	−0.0093 (4)
C2B	0.0267 (6)	0.0357 (7)	0.0207 (6)	0.0061 (5)	0.0059 (5)	−0.0043 (5)
C3B	0.0179 (5)	0.0285 (6)	0.0258 (6)	0.0027 (4)	0.0075 (4)	0.0016 (5)
C4B	0.0142 (4)	0.0144 (4)	0.0129 (4)	−0.0013 (3)	0.0005 (3)	−0.0001 (3)
C5B	0.0170 (4)	0.0145 (4)	0.0143 (5)	−0.0005 (3)	0.0006 (3)	0.0000 (3)
C6B	0.0277 (6)	0.0199 (5)	0.0278 (6)	0.0083 (4)	−0.0003 (5)	−0.0044 (4)
C7B	0.0218 (5)	0.0311 (6)	0.0238 (6)	0.0065 (4)	0.0022 (4)	−0.0093 (5)
C8B	0.0260 (6)	0.0360 (7)	0.0211 (6)	0.0051 (5)	0.0085 (5)	−0.0033 (5)
C9B	0.0179 (4)	0.0207 (5)	0.0143 (5)	−0.0006 (4)	0.0001 (4)	0.0015 (4)

### Geometric parameters (Å, °)

**Table:**

S1A—C1A	1.8171 (12)	S1B—C4B	1.8088 (11)
S1A—C4A	1.8330 (10)	S1B—C1B	1.8183 (12)
S2A—C4A	1.8119 (11)	S2B—C3B	1.8123 (13)
S2A—C3A	1.8227 (12)	S2B—C4B	1.8329 (10)
S3A—C6A	1.8185 (13)	S3B—C5B	1.8079 (11)
S3A—C5A	1.8207 (11)	S3B—C6B	1.8165 (13)
S4A—C5A	1.8099 (11)	S4B—C5B	1.8206 (11)
S4A—C8A	1.8162 (13)	S4B—C8B	1.8218 (13)
O1A—C9A	1.2013 (15)	O1B—C9B	1.2052 (14)
C1A—C2A	1.5222 (16)	C1B—C2B	1.5200 (19)
C1A—H1AA	0.9700	C1B—H1BA	0.9700
C1A—H1AB	0.9700	C1B—H1BB	0.9700
C2A—C3A	1.5193 (16)	C2B—C3B	1.524 (2)
C2A—H2AA	0.9700	C2B—H2BA	0.9700
C2A—H2AB	0.9700	C2B—H2BB	0.9700
C3A—H3AA	0.9700	C3B—H3BA	0.9700
C3A—H3AB	0.9700	C3B—H3BB	0.9700
C4A—C9A	1.5287 (15)	C4B—C9B	1.5197 (16)
C4A—C5A	1.5350 (15)	C4B—C5B	1.5333 (15)
C5A—H5AA	0.9800	C5B—H5BA	0.9800
C6A—C7A	1.5177 (19)	C6B—C7B	1.5159 (19)
C6A—H6AA	0.9700	C6B—H6BA	0.9700
C6A—H6AB	0.9700	C6B—H6BB	0.9700
C7A—C8A	1.5210 (19)	C7B—C8B	1.520 (2)
C7A—H7AA	0.9700	C7B—H7BA	0.9700
C7A—H7AB	0.9700	C7B—H7BB	0.9700
C8A—H8AA	0.9700	C8B—H8BA	0.9700
C8A—H8AB	0.9700	C8B—H8BB	0.9700
C9A—H9AA	0.9300	C9B—H9BA	0.9300
			
C1A—S1A—C4A	100.07 (5)	C4B—S1B—C1B	102.44 (5)
C4A—S2A—C3A	102.32 (5)	C3B—S2B—C4B	99.53 (5)
C6A—S3A—C5A	97.44 (6)	C5B—S3B—C6B	97.30 (6)
C5A—S4A—C8A	96.48 (5)	C5B—S4B—C8B	97.18 (6)
C2A—C1A—S1A	112.77 (8)	C2B—C1B—S1B	114.71 (9)
C2A—C1A—H1AA	109.0	C2B—C1B—H1BA	108.6
S1A—C1A—H1AA	109.0	S1B—C1B—H1BA	108.6
C2A—C1A—H1AB	109.0	C2B—C1B—H1BB	108.6
S1A—C1A—H1AB	109.0	S1B—C1B—H1BB	108.6
H1AA—C1A—H1AB	107.8	H1BA—C1B—H1BB	107.6
C3A—C2A—C1A	113.07 (9)	C1B—C2B—C3B	114.09 (11)
C3A—C2A—H2AA	109.0	C1B—C2B—H2BA	108.7
C1A—C2A—H2AA	109.0	C3B—C2B—H2BA	108.7
C3A—C2A—H2AB	109.0	C1B—C2B—H2BB	108.7
C1A—C2A—H2AB	109.0	C3B—C2B—H2BB	108.7
H2AA—C2A—H2AB	107.8	H2BA—C2B—H2BB	107.6
C2A—C3A—S2A	114.46 (9)	C2B—C3B—S2B	113.05 (9)
C2A—C3A—H3AA	108.6	C2B—C3B—H3BA	109.0
S2A—C3A—H3AA	108.6	S2B—C3B—H3BA	109.0
C2A—C3A—H3AB	108.6	C2B—C3B—H3BB	109.0
S2A—C3A—H3AB	108.6	S2B—C3B—H3BB	109.0
H3AA—C3A—H3AB	107.6	H3BA—C3B—H3BB	107.8
C9A—C4A—C5A	106.84 (8)	C9B—C4B—C5B	107.55 (9)
C9A—C4A—S2A	114.46 (8)	C9B—C4B—S1B	114.83 (8)
C5A—C4A—S2A	110.63 (7)	C5B—C4B—S1B	110.18 (7)
C9A—C4A—S1A	103.39 (7)	C9B—C4B—S2B	102.57 (7)
C5A—C4A—S1A	107.36 (7)	C5B—C4B—S2B	107.74 (7)
S2A—C4A—S1A	113.58 (5)	S1B—C4B—S2B	113.45 (6)
C4A—C5A—S4A	113.09 (7)	C4B—C5B—S3B	112.57 (7)
C4A—C5A—S3A	109.25 (7)	C4B—C5B—S4B	108.94 (7)
S4A—C5A—S3A	113.55 (6)	S3B—C5B—S4B	113.13 (6)
C4A—C5A—H5AA	106.8	C4B—C5B—H5BA	107.3
S4A—C5A—H5AA	106.8	S3B—C5B—H5BA	107.3
S3A—C5A—H5AA	106.8	S4B—C5B—H5BA	107.3
C7A—C6A—S3A	114.14 (8)	C7B—C6B—S3B	114.68 (9)
C7A—C6A—H6AA	108.7	C7B—C6B—H6BA	108.6
S3A—C6A—H6AA	108.7	S3B—C6B—H6BA	108.6
C7A—C6A—H6AB	108.7	C7B—C6B—H6BB	108.6
S3A—C6A—H6AB	108.7	S3B—C6B—H6BB	108.6
H6AA—C6A—H6AB	107.6	H6BA—C6B—H6BB	107.6
C6A—C7A—C8A	113.48 (10)	C6B—C7B—C8B	114.05 (11)
C6A—C7A—H7AA	108.9	C6B—C7B—H7BA	108.7
C8A—C7A—H7AA	108.9	C8B—C7B—H7BA	108.7
C6A—C7A—H7AB	108.9	C6B—C7B—H7BB	108.7
C8A—C7A—H7AB	108.9	C8B—C7B—H7BB	108.7
H7AA—C7A—H7AB	107.7	H7BA—C7B—H7BB	107.6
C7A—C8A—S4A	114.41 (10)	C7B—C8B—S4B	113.28 (9)
C7A—C8A—H8AA	108.7	C7B—C8B—H8BA	108.9
S4A—C8A—H8AA	108.7	S4B—C8B—H8BA	108.9
C7A—C8A—H8AB	108.7	C7B—C8B—H8BB	108.9
S4A—C8A—H8AB	108.7	S4B—C8B—H8BB	108.9
H8AA—C8A—H8AB	107.6	H8BA—C8B—H8BB	107.7
O1A—C9A—C4A	125.78 (11)	O1B—C9B—C4B	125.14 (11)
O1A—C9A—H9AA	117.1	O1B—C9B—H9BA	117.4
C4A—C9A—H9AA	117.1	C4B—C9B—H9BA	117.4
			
C4A—S1A—C1A—C2A	−61.63 (9)	C4B—S1B—C1B—C2B	−53.25 (11)
S1A—C1A—C2A—C3A	69.59 (12)	S1B—C1B—C2B—C3B	62.79 (14)
C1A—C2A—C3A—S2A	−65.11 (12)	C1B—C2B—C3B—S2B	−68.27 (13)
C4A—S2A—C3A—C2A	54.44 (10)	C4B—S2B—C3B—C2B	61.78 (10)
C3A—S2A—C4A—C9A	64.43 (9)	C1B—S1B—C4B—C9B	−62.56 (9)
C3A—S2A—C4A—C5A	−174.81 (7)	C1B—S1B—C4B—C5B	175.84 (8)
C3A—S2A—C4A—S1A	−54.00 (7)	C1B—S1B—C4B—S2B	54.96 (7)
C1A—S1A—C4A—C9A	−67.55 (8)	C3B—S2B—C4B—C9B	65.98 (8)
C1A—S1A—C4A—C5A	179.71 (7)	C3B—S2B—C4B—C5B	179.29 (8)
C1A—S1A—C4A—S2A	57.08 (7)	C3B—S2B—C4B—S1B	−58.47 (7)
C9A—C4A—C5A—S4A	−171.08 (7)	C9B—C4B—C5B—S3B	169.01 (7)
S2A—C4A—C5A—S4A	63.73 (9)	S1B—C4B—C5B—S3B	−65.16 (8)
S1A—C4A—C5A—S4A	−60.71 (8)	S2B—C4B—C5B—S3B	59.08 (8)
C9A—C4A—C5A—S3A	61.41 (9)	C9B—C4B—C5B—S4B	−64.66 (9)
S2A—C4A—C5A—S3A	−63.79 (8)	S1B—C4B—C5B—S4B	61.17 (8)
S1A—C4A—C5A—S3A	171.78 (5)	S2B—C4B—C5B—S4B	−174.59 (5)
C8A—S4A—C5A—C4A	170.50 (8)	C6B—S3B—C5B—C4B	−172.25 (8)
C8A—S4A—C5A—S3A	−64.27 (7)	C6B—S3B—C5B—S4B	63.71 (7)
C6A—S3A—C5A—C4A	−168.90 (7)	C8B—S4B—C5B—C4B	169.19 (8)
C6A—S3A—C5A—S4A	63.85 (7)	C8B—S4B—C5B—S3B	−64.81 (7)
C5A—S3A—C6A—C7A	−59.07 (10)	C5B—S3B—C6B—C7B	−59.23 (10)
S3A—C6A—C7A—C8A	64.76 (13)	S3B—C6B—C7B—C8B	64.90 (14)
C6A—C7A—C8A—S4A	−66.24 (13)	C6B—C7B—C8B—S4B	−65.45 (14)
C5A—S4A—C8A—C7A	61.09 (10)	C5B—S4B—C8B—C7B	60.64 (11)
C5A—C4A—C9A—O1A	−140.54 (12)	C5B—C4B—C9B—O1B	141.54 (11)
S2A—C4A—C9A—O1A	−17.70 (15)	S1B—C4B—C9B—O1B	18.52 (15)
S1A—C4A—C9A—O1A	106.35 (12)	S2B—C4B—C9B—O1B	−105.02 (12)

### Hydrogen-bond geometry (Å, °)

**Table:**

*D*—H···*A*	*D*—H	H···*A*	*D*···*A*	*D*—H···*A*
C2A—H2AB···O1A^i^	0.97	2.51	3.3530 (15)	146
C3A—H3AB···O1A	0.97	2.48	3.1024 (16)	122
C6A—H6AB···O1B^ii^	0.97	2.51	3.4292 (15)	159
C1B—H1BA···O1B	0.97	2.44	3.0508 (16)	121
C2B—H2BA···O1B^iii^	0.97	2.54	3.1913 (16)	124
C3B—H3BA···S2A^iv^	0.97	2.81	3.5932 (12)	138
C7B—H7BA···O1A^v^	0.97	2.54	3.3436 (17)	140

Symmetry codes: (i) −*x*+1, *y*+1/2, −*z*+3/2; (ii) *x*, −*y*+1/2, *z*+1/2; (iii) *x*, −*y*+1/2, *z*−1/2; (iv) −*x*+2, −*y*+1, −*z*+1; (v) −*x*+1, −*y*+1, −*z*+1.
